# NucleoCraft: The Art of Stimuli-Responsive Precision in DNA and RNA Bioengineering

**DOI:** 10.34133/bmef.0050

**Published:** 2024-09-17

**Authors:** Lu Yu, Liangxiao Chen, Deeksha Satyabola, Abhay Prasad, Hao Yan

**Affiliations:** School of Molecular Sciences and Center for Molecular Design and Biomimetics, The Biodesign Institute, Arizona State University, Tempe, AZ 85281, USA.

## Abstract

Recent advancements in DNA and RNA bioengineering have paved the way for developing stimuli-responsive nanostructures with remarkable potential across various applications. These nanostructures, crafted through sophisticated bioengineering techniques, can dynamically and precisely respond to both physiological and physical stimuli, including nucleic acids (DNA/RNA), adenosine triphosphate, proteins, ions, small molecules, pH, light, and temperature. They offer high sensitivity and specificity, making them ideal for applications such as biomarker detection, gene therapy, and controlled targeted drug delivery. In this review, we summarize the bioengineering methods used to assemble versatile stimuli-responsive DNA/RNA nanostructures and discuss their emerging applications in structural biology and biomedicine, including biosensing, targeted drug delivery, and therapeutics. Finally, we highlight the challenges and opportunities in the rational design of these intelligent bioengineered nanostructures.

## Introduction

In the evolving landscape of bioengineering, the precise manipulation of biomolecular architectures through stimuli-responsive mechanisms marks a frontier in structural biology and medicinal sciences. This approach enables the dynamic modulation of smart and adaptive materials, endowed with biological functionalities inspired by nature. Substantial efforts have been made in developing nanomaterials that respond mechanically to specific stimuli [1,2]. These include liposomes [3], polymeric micelles [4], silica nanoparticles [5], and magnetic nanoparticles [6], all of which demonstrate remarkable abilities to autonomously alter their behaviors in response to environmental triggers. These triggers include endogenous stimuli such as pH, enzyme, and microRNA (miRNA), as well as exogenous physical stimuli like light, temperature, and electric fields [7,8]. Common responses to these stimuli involve conformational changes (such as expansion or contraction and folding or unfolding), assembly or disassembly, degradation, phase transition (from gel to sol or solid to liquid), and the induction of motion. Such capabilities offer vast potential for applications in drug delivery, biosensing, diagnostics, and therapeutic interventions, showcasing remarkable advancement in stimuli-responsive technologies [9,10]. Despite these advancements, the development of new nanomaterials with high precision, safety, and specificity remains challenging due to the complex nature of cellular components and environments. Addressing these challenges, there is a growing focus on novel systems characterized by exceptional programmability, biocompatibility, and precision.

Nucleic acids, DNA, and RNA, with their intrinsically defined features and programmability via Watson-Crick base pairing at the nanoscale, serve as potent building blocks for the self-assembly and bottom-up construction of nanostructures and nanodevices, extending beyond their primary roles in the storage and decoding of genetic information [11,12]. These devices hold promise for the development of new biological parts, systems, and devices—either to redesign existing systems found in nature or to interact with natural processes in response to natural stimuli. The fields of DNA and RNA nanotechnology have experienced rapid development over recent decades, with considerably expanded potential for applications in nanomedicine, including in cancer treatment, disease diagnosis, immune therapy, and gene therapy [13–15].

In this review, we introduce the development of DNA and RNA nanotechnology and their underlying logic for a stimuli-responsive design that adapts and responds to environmental cues. Additionally, we provide a systematic review of the recent progress in stimuli-responsive DNA and RNA nanostructures. At the end of this review, we discuss the challenges and opportunities in the bioengineering of structural DNA and RNA nanotechnology.

## From Structures to Functions: Nucleic Acid Nanotechnology

**Fig. 1.**
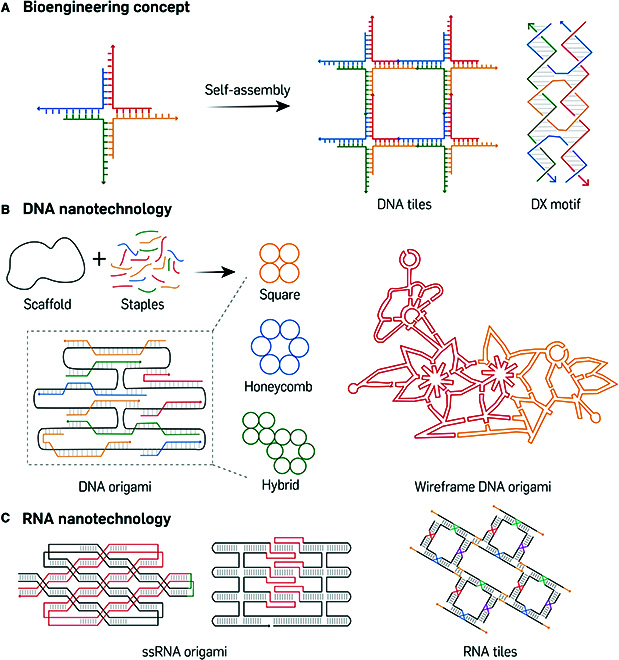
Structural nucleic acid nanotechnology.(A) Design of a DNA 2D lattice by self-assembly of an immobile 4WJ and DX motif. Adapted from Lin et al. [19] with permission. Copyright 2009, American Chemical Society. (B) The basic design principle for the self-assembly of DNA origami (left) and demonstration of the complex wireframe flower-and-bird DNA origami (right). Adapted from Zhang et al. [26] with permission. Copyright 2015, Springer Nature Limited. (C) Schematic of single-stranded DNA origami routing (left); adapted from Han et al. [28] with permission. Copyright 2017, The American Association for the Advancement of Science (AAAS). Schematic of KL based single-stranded RNA origami (center); adapted from Geary et al. [154] with permission. Copyright 2014, AAAS. Design principle of KL based RNA tile assembly (right); adapted from Chworos et al. [37] with permission. Copyright 2004, AAAS.

Nucleic acid nanotechnology involves the creation of DNA or RNA structures at the nanometer scale that form specific geometries and execute diverse functions. Since the discovery of the crystal structure of DNA in 1951, scientists have sought to create artificial nucleic acid constructs capable of specific functionalities. Inspired by naturally occurring 4-way “Holliday” junctions (4WJ) (Fig. 1A) that are critical intermediates in genetic recombination processes [16], Nadrian Seeman first proposed the concept of double crossover (DX) “tiles” in 1993 [17,18,19], which contained 2 immobilized 4WJ motifs to facilitate strand migration between adjacent helices, and his vision effectively laid the foundation for nucleic acid nanotechnology [20]. Based on the DX motif, a variety of self-assembling tile structures composed of multiple oligonucleotides were developed for the formation of discrete particles or large-scale structures [21,22]. In 2006, the design of DNA origami was introduced [23], which required a long single-stranded scaffold paired with multiple short complementary “staple” strands arranged in a predetermined pattern, to enable the formation of intricately shaped structures on the nanoscale (Fig. 1B) [24,25,26]. Concurrently, the single-stranded tile (SST) method emerged, which employed short oligonucleotides to create DNA nanostructures without the constraints of scaffold length, to facilitate the synthesis of nanostructures composed of over 10,000 strands [27]. Additionally, single-stranded origami was introduced to simplify the complexity of the system, to enable the encoding of structural information within a single-stranded sequence, emulating protein folding [28].

Parallel design principles have been successfully adapted to RNA, leading to the development of RNA tiles [29] and RNA origami (Fig. 1C) [30]. Meanwhile, RNA contains natural interaction motifs between stem–loops and bulges, including kissing loop [31], tectoRNA [32], and pRNA [33]. These elements offer enhanced design flexibility, which allows for the creation of RNA nanostructures with complex geometries (Fig. 1C) [34–37]. Unlike sticky ends, these interactions occur between closed strands and permit the design and self-assembly of single-stranded RNA nanostructures [28,38].

**Fig. 2.**
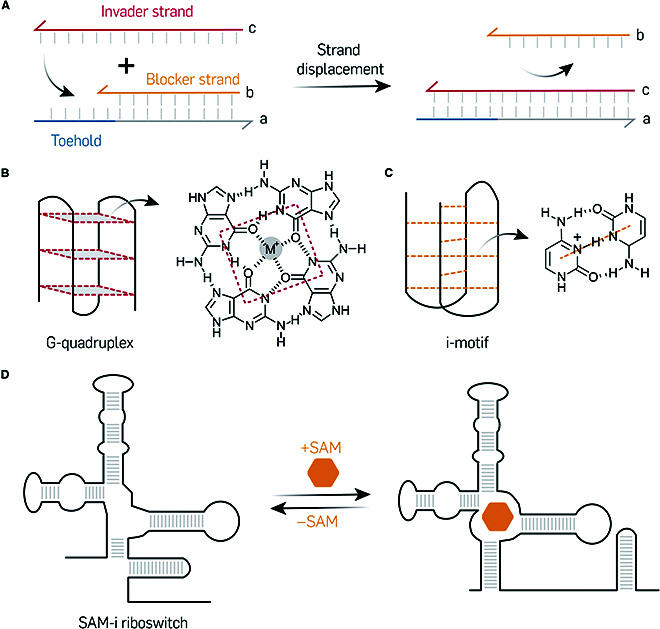
Responsive nucleic acid-based components in DNA and RNA nanostructures. (A) Demonstration of the strand displacement reaction. The image was adapted from Soloveichik et al. [42]. (B) Schematic of G-quadruplex. The image was adapted under a Creative Commons Attribution License and is attributed to Capra et al. [43]. (C) Schematic of i-motif. Adapted from Dong et al. [44] with permission. Copyright 2014, American Chemical Society. (D) Schematic of SAM-i riboswitch. The image was adapted under a Creative Commons Attribution License and is attributed to Huang et al. [47].

Nucleic acid nanostructures have great potential as smart materials in therapeutic applications due to their pronounced responsiveness [39]. Numerous functional motifs can be engineered to exhibit structural variability and respond to a variety of input signals, including DNA/RNA strands, small molecules, and ions. By integrating responsive nucleic acid-based components, it becomes possible to develop intelligent nanostructures that react to specific inputs, thereby offering great potential for targeted therapeutic applications [40]. One key mechanism underlying this adaptability is the strand displacement reaction (Fig. 2A) [41,42]. This process involves one strand “invading” a duplex and displacing 1 of 2 strands called “blocker”, a process that is driven by increased base pairing and reduced free energy of the final structure. The process affords dynamic control and functionality of nucleic acid nanostructures, making them acutely responsive to specific DNA or RNA strand sequences. Additionally, nucleic acid nanostructures can incorporate functional secondary structures. Common examples include the G-quadruplex (Fig. 2B) [43], which reacts to metal ions, and the i-motif (Fig. 2C) [44,45], which is sensitive to variations in pH. Riboswitches (Fig. 2D) [46,47] are naturally occurring regulatory segments found in the 5′ untranslated region (UTR) of mRNA, which can respond to small-molecule effectors by altering their secondary structure for the regulation of mRNA translation. Similarly, numerous artificial nucleic acid aptamer [48] sequences are screened to bind selectively to specific targets, including small molecules, proteins, and cells. Moreover, nucleic acids can be easily modified with various types of functional groups such as small molecules, lipids [49], dyes [50], and proteins [21]. This versatility allows for the formation of supramolecular structures to serve as platforms for these functional groups, enabling them to respond to diverse stimuli.

Together, these design principles have equipped researchers with a rich toolkit for the construction and synthesis of responsive nanostructures of varied geometries and shapes [51].

## Stimuli-Responsive DNA Nanostructures

The programmability, biocompatibility, and availability of self-assembled DNA nanostructures, along with their highly controlled physical and chemical properties, such as size, shape, and surface modifications, render them ideal for biomedical applications, including diagnostics, targeted drug delivery, and therapeutics [13]. Beyond designing intricate static structures, the fabrication of dynamic structures that exhibit tailored conformational changes or drug release mechanisms in response to external stimuli further expands their utilization in precision biomedicine [52]. Generally, DNA nanostructures are constructed using 3 methods: (a) tile-based assembly, (b) DNA origami, and (c) supramolecular-based assembly [53].

### Stimuli-responsive tile-based DNA nanostructures

DNA tile-based self-assembly, defined as a bottom-up approach where engineered DNA structures (tiles) organize into a predefined architecture, is a fundamental method in DNA nanotechnology. These assemblies, based on programmable nucleotide sequences, form building blocks for one-dimensional (1D) and 2D arrays and 3D nanostructures [22,54]. Although most of these systems are initially very rigid and static, the field has recently overcome these limitations by designing dynamic, stimuli-responsive tile-based DNA nanostructures [55], which can adapt and change their configuration in response to specific environmental triggers. This remarkable achievement marks a remarkable evolution in the application and functionality of DNA-based assembly [52].

**Fig. 3.**
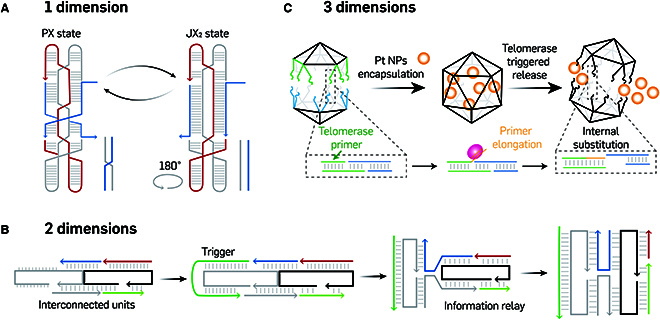
Stimuli-responsive tile-based DNA nanostructures. (A) A reconfigurable PX-JX2 rotary DNA device. Adapted from Yan et al. [56] with permission. Copyright 2002, Macmillan Magazines Ltd. (B) Information relay from one antijunction tile to neighbors with the addition of trigger strand. Adapted from Song et al. [58] with permission. Copyright 2017, AAAS. (C) Telomerase responsive DNA icosahedron. Adapted from Ma et al. [64] with permission. Copyright 2018, Wiley-VCH.

In 2002, Yan et al. [56] innovatively combined strand displacement with structural self-assembly to develop a dynamic, reconfigurable rotary DNA device operating through a 4-stage cyclic process (Fig. 3A). This device utilized the toehold-mediated strand displacement (TMSD) technique to enable interconversion between the paranemic crossover (PX) motif and its topoisomer, the juxtaposed (JX_2_) motif, wherein one strand end rotates 180° relative to the other end. By controlling the addition of “fuel” (fully complementary to the set strand) and the “set” strand (which sets the state of the structure), the device transitioned between the *cis* arrangement (PX state) and the zigzag *trans* arrangement (JX_2_ state). More recently, Yang and colleagues designed extensive 1D and 2D arrays using a series of T-shape crossover (TC) tiles. These arrays combined the structural attributes of the antiparallel double crossover (AX) tile for enhanced rigidity with the T-junction tile for precise angle and orientation control [57]. They further engineered 2-state reconfigurable nanorings from C-shape TC tiles. Here, DNA strands acted as triggers to facilitate geometric reconfigurations, introducing functional toeholds in the horizontal arm of the C-shaped TC tiles by varying the arm lengths with the invader and setting strands, leading to curvature transformations. These innovative, stimuli-responsive nanorings can enlarge or contract, mimicking the geometric changes of nanopores, which offer motivating opportunities for constructing artificial, stimuli-responsive DNA nanopores.

A transformation cascade is crucial in biological processes. Song et al. [58] replicated these complex dynamic behaviors in a highly controlled DNA array system, resembling a molecular “domino array” (Fig. 3B). They designed a reconfigurable DNA relay array from dynamic DNA anti-junction tiles, which contain 4 double helices and 4 dynamic nicking points. These enabled a conformational switch between 2 stable states though an unstable open state. The addition of trigger DNA strands, which removed a mobile nick from the anti-junction unit at selected positions in the array, induced a conformational switch from one anti-junction unit to its neighbor, which propagated throughout the array, thus obviating the need for additional triggers. This stepwise information relay was driven by base stacking, wherein the conformational change of one anti-junction creates a high-energy interface and weakened base stacking between the transformed unit and its neighbor.

Stimuli-responsive tile-based DNA nanostructures can be engineered to regulate or be regulated by enzymatic reactions. Liu et al. [59] developed a tweezer-like DNA nanoreactor, self-assembled from 2 DNA DX motifs connected by a Holliday junction, capable of toggling the activity of enzyme/cofactor pair on and off. In this system, glucose-6-phosphate dehydrogenase (G6PDH) and the NAD^+^ cofactor were attached to the different arms of the DNA tweezer in an open state that spatially inhibited activity. The enzyme was activated by adding a fuel strand that transitioned the structure from open to closed, thus bringing the cofactor into proximity with the enzyme. More recently, Farag et al. [60] constructed enzyme-responsive, LEGO-like tubular DNA nanostructures from antiparallel double-crossover DNA tiles (DAE-E). This design enabled dynamic and reversible control over the assembly, effectively preventing component crosstalk. The control mechanism involved enzymatically degradable, label-conjugated strands that dissociated upon degradation by RNase H or uracil-DNA glycosylase (UDG), facilitating the replacement with new, unique strands. These developments showcase a promising direction for creating functional biomaterials with enhanced precision, adaptability, and sensing capabilities.

Engineered 3D nanostructures assembled from multi-arm junctions have shown excellent properties for active targeting and on-demand drug release, crucial for effective drug delivery systems. In 2016, Bujold et al. [61] introduced a DNA “nanosuitcase”, a trigger-responsive, siRNA-encapsulating prism that selectively releases its cargo in response to specific oligonucleotide triggers in complex cellular environments, demonstrating potential for targeted and controlled drug delivery. Similarly, Tian and co-workers developed a stimuli-responsive dynamic DNA tetrahedron that responded to environmental stimuli such as proteins and pH, which differ in concentration between cancerous and normal environments. This responsiveness led to the tetrahedron’s disassembly and subsequent drug release [62]. The tetrahedron featured an AS1411 aptamer on one edge for specific cell membrane targeting and a cytosine-rich sequence, folded into a pH-responsive quadruplex i-motif, aimed at the acidic tumor microenvironment (TME). To encapsulate drugs, 2 strategies were used: one where the drug bound to DNA overhangs directed inward, and another where they were secured inside the tetrahedron’s cavity by overhangs on opposite sides of the exoskeleton.

To increase the complexity and size of tile-based 3D DNA nanostructure, Banerjee and colleagues developed a cyclic-di-GMP (cdGMP)-responsive DNA icosahedron, which incorporated cdGMP aptamers and disassembled into 2 constituent halves through strand displacement, thereby releasing its encapsulated cargo [63]. Similarly, Gu’s group engineered a telomerase-responsive DNA icosahedron designed to release encapsulated platinum nanoparticles (PtNPs) specifically in cisplatin-resistant cancer cells with high telomerase activity, offering an effective therapy [64] (Fig. 3C). This icosahedron, constructed from 2 pyramidal DNA cages self-assembled from 3 sets of 5-arm junction tiles and connected via telomerase primers and telomeric repeats, disassembled upon exposure to telomerase, releasing the encapsulated PtNPs. Furthermore, Wang et al. advanced the field with smart DNA logic devices that distinguish various cellular conditions for controlled stimuli responsiveness. Their environment-responsive DNA computation circuits, integrating adenosine triphosphate (ATP), H^+^, and mRNA inputs, utilized a truncated square pyramid with an antisense DNA strand for mRNA targeting, an ATP aptamer, and an i-motif [65]. These components functioned as logic-control modules (OR–AND and AND–AND), responding to endogenous signals via TMSD reactions to control the release and dissociation of duplex cargo. This logic-controlled, stimuli-responsive, 3D nanostructure represented a novel approach to smart precision medicine.

### Stimuli-responsive DNA origami

The introduction of DNA origami has revolutionized structural DNA nanotechnology, marking an important shift from tile-based assemblies to a method capable of creating highly complex and arbitrarily designed 2D and 3D nanostructures [23]. This approach facilitates the precise folding of DNA strands into a myriad of predetermined shapes and patterns. By integrating DNA origami with dynamic reconfiguration capabilities that respond to various physiological stimuli, the technology offers remarkable versatility and modularity. Consequently, it can facilitate the automatic execution of complex nanomechanical tasks and open new opportunities for biomedical applications.

An autonomous aptamer-gated DNA origami nanorobot, designed as a hexagonal barrel, has been used for transporting molecular cargo to specific cell types. The system was controlled by a DNA aptamer-encoded logic gate, allowing it to reconfigure its structure for payload delivery in response to cell surface antigens [66]. It was composed of 2 domains linked by scaffold hinges, with aptamer-complement duplexes incorporated on both sides of the front of the barrel to control the open and closed states of the DNA cage. This aptamer-encoded logic gate enhanced selectivity by requiring 2 inputs to open, functioning like an “AND” gate, and has been used to deliver antibody Fab fragments to targeted cells, thus stimulating cell signaling. Subsequently, a DNA vault (DV) that self-assembled from a honeycomb lattice and was regulated by the multi-lock system was developed to control enzyme–substrate interactions. Unlike the aptamer-protein logic gate, this reversible mechanism relied on the TMSD reaction [67]. When immersed in a substrate solution, the closed DV restricted substrate diffusion to the encapsulated enzyme. Upon the addition of opening keys, the DV underwent a conformational change from closed to open, activating the enzyme. Additionally, pH-responsive DNA motifs, known as Hoogsteen-type polypurine-polypyrimidine triplexes, were used in the locking system of multi-layer reconfigurable DNA origami nanocapsules [68]. These “pH latches” managed reversible opening and closing cycles, forming a parallel triplex DNA in acidic conditions or hanging freely in basic conditions. Different types of cargo, such as gold nanoparticles (AuNPs) and horseradish peroxidase (HRP), were loaded into the cavities of these nanocapsules.

**Fig. 4.**
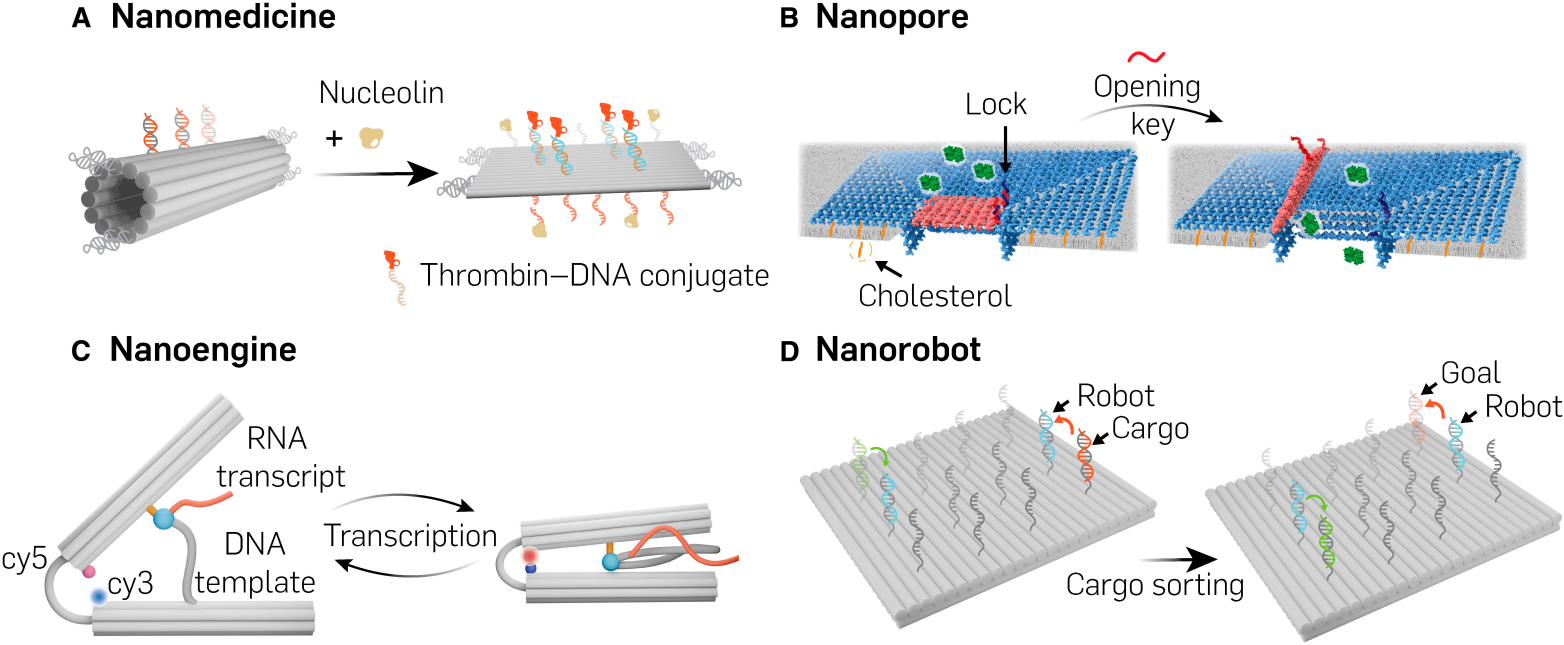
Stimuli-responsive DNA origami nanostructures. (A) Design of a thrombin loaded nucleolin-responsive DNA nanomedicine for cancer therapy. Adapted from Li et al. [69] with permission. Copyright 2018, Springer Nature. (B) Design of reconfigurable nanopore, LGC-C and LGC-O for protein transport regulation. Adapted from Dey et al., under the terms of Creative Commons CC BY license [75]. (C) Design of a rhythmically pulsing DNA origami leaf-spring nanoengine. The image was adapted under the CC BY license and is attributed to Centola et al. [80]. (D) Cargo sorting DNA nanorobots on the DNA origami testing ground. Adapted from Thubagere et al. [81] with permission. Copyright 2017, AAAS.

Subsequently, Li’s group demonstrated the first in vivo bioapplication of trigger-responsive DNA origami for targeted cancer therapy [69]. They developed an intelligent DNA nanorobot designed to deliver thrombin specifically to tumor-associated blood vessels in tumor-bearing mice, inducing tumor necrosis and inhibiting tumor growth in response to tumor vessel marker nucleolin (Fig. 4A). This autonomous DNA nanorobot was self-assembled from a rectangular DNA origami sheet. After thrombin loading, DNA aptamers, which specifically bind to nucleolin protein, were used as fastener strands to bring the first and last helices of the DNA nanosheet together, resulting in the formation of a tubular DNA origami structure with well-defined inner and outer protective surfaces for the loaded protein. The transformation from tubular shape back to rectangular to expose the protected thrombin was triggered by nucleolin protein binding. The biodistribution, biosafety, and antitumor efficacy of the DNA nanorobot have been extensively evaluated, highlighting its potential as potent nanomedicine. Yang’s group further developed a thrombin concentration-triggered DNA nanorobot from a barrel-shaped origami framework with an embedded molecular reaction cascade computing core comprising an input sensor, a threshold controller, and inhibitor functional modules. This barrel-shaped DNA origami assembly responded to precise thrombin levels, which offers prospects for autonomous anticoagulation and personalized nanomedicine [70]. Controlled drug release of drug delivery nanoplatform in vivo is crucial for cancer therapy. Zhang et al. reported a pH-controlled drug release system using a triangular DNA origami nanodevice. The anticancer drug doxorubicin, loaded through intercalation, was efficiently released from the drug delivery vehicle in acidic tumor regions and subcellular organelles, demonstrating prominent anticancer efficacy in vivo [71]*.*

Recently, Engelen et al. [72] reported a new logic-gating DNA nanodevice triggered by antigens. They designed an antigen-responsive, reconfigurable icosahedral DNA origami shell assembled from 20 identical triangular DNA origami subunits using shape-complementary blunt-end stacking. This structure was kinetically trapped in metastable stats by bivalent immunoglobulin G antibodies, which acted as structural lockers with 2 pairs on each edge. Upon exposure to free antigens, antibody-stapled shells can burst and disassemble into triangular monomers. Additionally, an “AND” logic gate has been integrated into this system, which allows it to respond to 2 distinct antigens simultaneously. As a proof of concept, the system was used to demonstrate antigen-triggered release by encapsulating hepatitis B virus (HBV) core particles within the shell cavity, showing its potential for controlled drug delivery. In addition, Zhang and co-workers expanded the capabilities of DNA nanodevices by employing in-site synthesis of DNA origami in complex environments responsive to NIR photothermal conversion. This technique is highly precise and efficient, both in cell lysate and in cell culture settings, opening up possibilities for applications in deep tissue responsiveness and cancer theranostics [73]. Kohman and Han [74] developed a large-scale, reconfigurable DNA nanosphere that was responsive to light irradiation. This structure incorporated 9 photo-cleavable spacers (*ortho*-nitrobenzyl, *o*-NB) at the staple crossovers that connected the 2 hemispheres at the sphere’s equator. Upon light irradiation, these photo-cleavable crossovers broke apart, allowing the unfolded excess scaffold DNA, which acted as a hinge, to enable the hemispheres to separate by approximately 100 nm. Collectively, these advancements highlight the transformative potential of DNA origami in creating responsive and adpatable nanostructures for diverse biomedical applications.

Dey et al. [75] developed a novel DNA nanopore, termed the “large and gated channel” (LGC), which employed a “horizontal” routing strategy distinct from traditional “vertical” artificial DNA nanopores (Fig. 4B). This design allows for the customization of pore size based on functional needs. The LGC was composed of single-layer DNA origami with large external dimensions for cholesterol anchoring, facilitating its insertion into membranes or puncturing through membrane bilayers. It featured a cross-sectional square-channel lumen that enables cargo transport. For reversible operation, the LGC utilized a square lid constructed from horizontally routed DNA duplexes, attached on one side to the channel plate by 2 flexible hinges. The opposite side contained 2 single-stranded half-locks that hybridized with complementary counterparts at the channel base, enabling switchable transport control via a TMSD reaction. This mechanism effectively regulated the flux of fluorescent substances like Atto633 and green fluorescent protein (GFP) across the bilayer. Similarly, Wang et al. [76] previously designed a DNAzyme- and/or light-responsive nanohole also using a DNA origami nanoplatform. This device featured engineered “window” domains locked by either Zn^2+^- or Pb2^+^-ion-dependent DNAzymes, or by photoisomerizable azobenzene-modified strands. This design showcased versatility, potentially enhancing a variety of biocatalytic cascades and cell membrane channel control. The self-catalytic capability of DNAzymes under various conditions, combined with their potential in biosensing and diagnostics, makes them ideal for integration into the design of stimuli-responsive DNA nanostructures for multiple biomedical applications [77,78]. By incorporating DNAzymes, these nanostructures can be engineered to respond to specific environmental triggers, enabling controlled activation or deactivation of their catalytic function and enhancing their adaptability and functionality in targeted drug delivery, biosensing, and nanomedicine. Shi et al. [79] developed a one-stranded allosteric catalytic DNAzyme-based nucleic acid biosensor, named SPOT (sensitive loop-initiated DNAzyme biosensor for nucleic acid detection). SPOT comprised a catalytic core, 2 fully locked flanking arms, a single-stranded loop that served as the allosteric module for nucleic acid detection. Nucleic acid targets continuously bound to the allosteric loop region, resulting in continuous cleavage for signal amplification. The authors demonstrated that SPOP could detect serum miRNAs with high sensitivity and specificity for the diagnostic of multiple cancers, as well as for SARS-CoV-2 RNA detection. SPOT was also adaptable to other testing modalities. Therefore, integrating such DNAzyme-based systems into the construction of stimuli-responsive nanostructures will enhance the potential for precise and responsive biomedical applications.

More recently, a DNA origami-based nanoengine that can pulsate rhythmically, fueled by nucleoside triphosphates (NTPs), has been reported (Fig. 4C). This development further expanded the capability of intelligent DNA nanorobots to perform complex and sophisticated tasks. The rhythmic pulsating DNA leaf-spring nanoengine was assembled from 2 origami arms joined by a DNA hinge [80]. The arms were linked on the opposite side by a double-stranded DNA template (dsDNA-t) that included a T7 promoter sequence, an arbitrary sequence, and a T7 terminator sequence. T7 RNA polymerase was covalently attached adjacent to the dsDNA-t on one of the DNA origami arms. In the presence of NTPs, the transcription of the dsDNA-t was initiated, pulling the 2 arms together and transferring this motion to its passive moving parts. This movement was released after the RNA synthesis was completed, and the process continued to repeat, thus inducing the rhythmic pulsing motion phenomenon.

Designing molecular nanomachines that could autonomously perform intricate nanomechanical tasks and interact with their environments posed substantial challenges in molecular engineering, particularly in maintaining modularity and simplifying algorithms. Thubagere et al. [81] have made strides in this field with the development of a cargo-sorting DNA nanorobot composed of 3 modular building blocks. This robot was capable of executing random walking and adept at retrieving and delivering cargo, utilizing the TMSD reaction for precise molecular-level cargo sorting (Fig. 4D). Engineered to remain stationary initially, the robot was activated when unit trigger strands were introduced, which disengaged an inhibitor, setting the robot in motion. This design was streamlined for simplicity, featuring a single-stranded DNA body with a leg and 2 feet domains for movement, alongside an arm and a hand domain for handling cargo. Operating on a 2D origami testing surface, the robot efficiently gathered various cargos and transported them to predetermined destinations, sorting them at specific locations. This demonstration marked a outstanding advancement in the functionality and application of DNA-based nanorobots.

### Stimuli-responsive supramolecular DNA assembled nanostructures

The integration of DNA building blocks with synthetic organic, inorganic, and polymeric molecules marked a prominent advance in DNA nanotechnology, facilitating the development of responsive and adaptable nanostructures for biological and medical applications [53]. This evolution to supramolecular DNA nanostructures, which enhanced flexibility and responsiveness, was characterized by their assembly through diverse non-covalent interactions like hydrogen bonding, electrostatic forces, and hydrophobic interactions. These systems are notable for their ability to dynamically reconfigure in response to environmental stimuli and incorporate a variety of building blocks that lead to more complex, multifunctional, and heterogeneous structures. This pivotal transition in the field not only overcame the chemical limitations of traditional DNA nanostructures but also broadened the scope for the creation of higher-order structures.

**Fig. 5.**
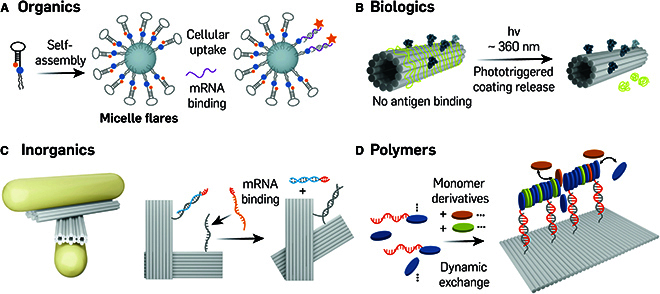
Stimuli-responsive supramolecular DNA assembled nanostructures. (A) Lipid integrated mRNA responsive MBMFs for mRNA detection and gene therapy. Adapted from Chen et al. [85] with permission. Copyright 2013, Wiley-VCH. (B) Protein-coated photoreversible supramolecular DNA nanostructures. The image was adapted under a CC BY 4.0 license and is attributed to Seitz et al. [87]. (C) An optical responsive 3D gold-DNA hybrid structure for targeted RNA detection. Adapted from Funck et al. [89] with permission. Copyright 2018, Wiley-VCH. (D) Dynamic assembly of supramolecular polymers on a DNA origami nanoplatform. The image was adapted under a CC BY-NC license and is attributed to Schill et al. [90].

In 1996, Chad Mirkin’s group first introduced spherical nucleic acid (SNA) using gold nanoparticle templates and short DNA strands [82], a discovery that has gained increasing interest over the past 2 decades, with various types of nanoparticle cores and nucleic acids utilized for diverse applications [83]. Fakih et al. [84] developed a stimuli-responsive, component-minimal SNA capable of conditionally silencing target genes in living cells in response to 2 cytoplasmic genetic markers. This SNA comprised a hydrophobic core and a DNA-based corona, within which 2 strands were partially hybridized using a “bridge” strand. The therapeutic oligonucleotide was hybridized onto the horizontal portion of the bridge, enabling its release in the presence of fully complementary strands (2 triggers) through a TMSD mechanism. Based on a similar strategy, Chen et al. [85] developed mRNA-responsive molecular beacon micelle flares (MBMFs) with a diacyllipid core and a molecular beacon (MB) corona (Fig. 5A). These MBMFs, formed by the spontaneous self-assembly of diacyllipid-beacon conjugates (L-MBs) via hydrophobic interactions, were designed for *c-raf-1* mRNA targeting to facilitate gene silencing, incorporating a quencher–fluorophore pair at its ends for detection. In the absence of targeted mRNA, the MEMFs operated in an OFF state, where the fluorophore and quencher were in close proximity. Upon recognition of the targeted mRNA, the MBMFs underwent a conformational change that resulted in the hairpin opening, separating the fluorophore and quencher and shifting the MBMFs to an ON state, enabling fluorescence emission upon excitation. Subsequently, mRNA degradation was induced by the RNase enzyme, resulting in the inhibition of *c-raf-1* mRNA expression, which could potentially impede tumor progression.

Recently, there has been increasing interest in supramolecular co-assembled DNA nanostructures, which were integrated electrostatically with other biological molecules such as cationic lipids and positively charged proteins [86]. These assemblies were designed to respond to external stimuli, thereby improving stability and facilitating multi-functionalization for biomedical applications. Seitz and co-workers developed a light-responsive, 2-component protein coating technique that outfitted rod-shaped, 24-helix bundle DNA origami with an anti-HER2 antibody for targeting and bovine serum albumin (BSA) protein for camouflaging via electrostatic interactions [87] (Fig. 5B). The positively charged DNA-binding domain in each dendron branch of the BSA–dendron complex contained a photolabile group. Upon exposure to light, these groups underwent cleavage from the binding domain, leading to their dissociation from the structure and exposing the anti-HER2 antibody fragment for triggered binding to the antigen. This approach demonstrated the potential for highly controlled, protected, and targeted drug delivery. More recently, Julin et al. [88] reported on the development of light-responsive, highly ordered lipid-DNA fibers forming a 2D hexagonal lattice. These supramolecular DNA nanostructures were co-assembled from rod-shaped, 6-helix bundle (6HB) DNA origami and cationic, photosensitive lipids via electrostatic and hydrophobic interactions. Upon UV light irradiation, the photosensitive groups attached to the lipid were cleaved, facilitating the disassembly of the co-assembled supramolecular structures by removing the lipid tails. This eliminated the hydrophobic driving force essential for the multivalent co-assembly, resulting in the release of 6HBs. This approach presents a sophisticated strategy for designing responsive nanostructures with potential applications in therapeutics.

Inorganic molecules like gold nanorods (AuNRs) can also be incorporated into DNA nanostructures to introduce a plasmonic chiral response into the supramolecular system. Funck et al. [89] constructed a 3D gold–DNA hybrid structure, containing 2 AuNR-modified DNA origami arms connected by 2 single-stranded DNA oligonucleotides in the center that allowed for the flexible orientation of the arms to enable sensitive detection of specific RNA in human serum samples using a chiral plasmonic switch (Fig. 5C). This conformational change was triggered by the binding of targeted RNA strands to the sensor region of the nanostructures. The sensor region consisted of 2 complementary DNA handles located on one end of each arm, which can lock the structure in a right-handed state. Initially, one of the DNA handles was hybridized with a blocking strand to prevent the locking of the structure; in the presence of the targeted RNA, the blocking strands were displaced via a TMSD reaction, resulting in the locked state. Consequently, this conformational change led to the twisted arrangement of nanorods, which produced a strong circular dichroism signal.

The incorporation of polymeric molecules can further increase the diversity of supramolecular stimuli-responsive DNA nanostructures in both structural design and functionality. Schill and colleagues reported on a hybrid DNA origami-based nanoplatform designed for dynamically templating the assembly of multicomponent, 1D supramolecular assemblies. This platform featured the site-selective recruitment of supramolecular polymers that preserve the intrinsic dynamics and rearrangements of the assembly process (Fig. 5D) [90]. The supramolecular system was assembled on the DNA origami template containing bipyridine-based C_3_-symmetrical amphiphilic discotic molecules. This assembly utilized a library of monomers with functionalized in various ways, including insert discs, DNA discs, dye-labeled discs, and cargo-loaded discs, to allow them to form intrinsically fluorescent columnar aggregates. Rectangular DNA origami equipped with handles complementary to the DNA-functionalized disc monomers served as the nanoplatform for recruiting these supramolecular assemblies. A deliberate design placed adjacent handles at 6 nm to accommodate non-DNA functionalized monomers. The non-covalent interactions inherent in these supramolecular assemblies facilitated dynamic tuning and rearrangement of assembly composition. This entails a dynamic and reversible exchange of monomeric components within the aggregates assembled on the DNA origami platform.

**Fig. 6.**
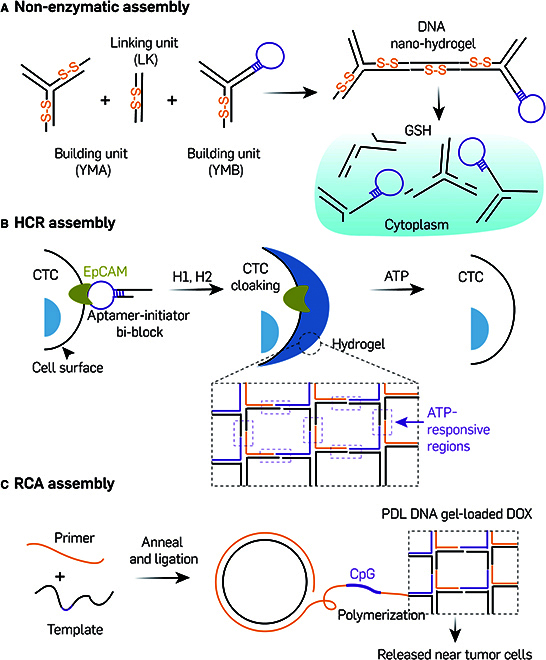
Stimuli-responsive DNA hydrogel. (A) A GSH-responsive, non-enzymatic, dendritic DNA cross-linking-based DNA hydrogel for gene regulation therapy. Adapted from Li et al. [94] with permission. Copyright 2015, American Chemical Society. (B) HCR-based self-assembled ATP-responsive DNA hydrogel for circulating tumor cell capturing. Adapted from Song et al. [97] with permission. Copyright 2017, American Chemical Society. (C) RCA-based self-assembled TME-responsive aPDL1/DOX@DNA hydrogel for chemo-immunotherapy. The image was adapted under a CC BY license and is attributed to Fan et al. [99].

Further extending the scope of stimuli-responsive, self-degradable supramolecular DNA assembled nanostructures, DNA hydrogels have emerged as a notable subclass within this domain for application due to their high drug loading capacity, programmability, and multifunctionality [91]. DNA hydrogels are 3D, mesh-like structures formed through either physical or chemical cross-linking that utilize DNA as either the primary structural backbone or cross-linking agents, and they possess the combined characteristics of conventional hydrogels and nucleic acids. Modulation of the cross-linking density of DNA hydrogels or incorporating functional materials allows for precise control over their size, biodegradability, and responsiveness to various stimuli [92]. There are 3 primary strategies for assembling DNA hydrogels: enzyme-catalyzed or non-enzymatic dendritic DNA cross-linking, hybridization chain reaction (HCR), and rolling circle amplification (RCA). The first enzyme-catalyzed DNA hydrogel synthesized using pure dendritic DNA was reported in 2006 [93]. Later, Li et al. [94] developed a non-enzymatic DNA hydrogel that was responsive to endogenous glutathione (GSH). This was based on 3 types of building units connected by sticky ends: Y-shaped monomer A (YMA), an aptamer-containing Y-shaped monomer B (YMB), and a DNA linker (LK) (Fig. 6A). Its size could be controlled by varying the ratio of YMA to YMB, and disulfide linkages that were incorporated into these building blocks were able to confer GSH stimuli responsiveness. Furthermore, therapeutic oligonucleotides and DNAzyme oligonucleotides were integrated in YMA and LK to target cancer-related mRNA and proteins. Consequently, a multifunctional, aptamer-based DNA hydrogel was created, featuring controllable size, specific cell targeting, stimuli-responsive disassembly, and therapeutic efficacy. The isothermal, enzyme-free HCR [95], which employs 2 hairpins to store potential energy, and can be initiated by a specific trigger to undergo a cascade chain reaction, is also frequently utilized to direct the assembly of stimuli-responsive 3D hydrogels [96]. Song et al. [97] applied the HCR technique for the in situ construction of an ATP-responsive DNA hydrogel. This hydrogel was based on an aptamer-initiated mechanism, designed for the capture and release of circulating tumor cells (Fig. 6B). The isothermal enzymatic amplification method known as RCA is another common strategy for synthesizing stimuli-responsive DNA hydrogels [98]. Fan et al. [99] reported the development of a TME-responsive chemo-immunotherapeutic DNA hydrogel (Fig. 6C), which incorporated CpG oligonucleotides and ATP aptamers encoded within ultralong DNA building blocks synthesized via RCA-mediated DNA polymerization. aPDL1 antibody, an immune checkpoint inhibitor, and the chemotherapeutic agent DOX were then sequentially incorporated into the DNA hydrogel through electrostatic interactions and intercalation, respectively. Triggered by ATP, the aptamers underwent a dramatic conformational change that resulted in the release of DOX from the gel. This event induced immunogenic cell death, which, together with CpG, recruited and activated dendritic cells leading to the sustained release of aPDL1 antibody, which further sensitized T-cell immunity and demonstrated that aPDL1/DOX@DNA hydrogel is an excellent synergistic therapeutic modality.

## Stimuli-Responsive RNA Nanostructures

RNA has also emerged as a versatile and potent building block for the construction of nucleic acid nanostructures. The innate complexity of RNA’s tertiary structures not only enriches the design possibilities but also enhances the responsiveness of these systems to various stimuli. Beyond its structural role, it plays an integral part in biological processes, such as protein synthesis via mRNA, and presents unique opportunities for biofunctional integration. By embedding responsive elements within RNA sequences, particularly mRNA, it becomes feasible to intricately manipulate biological functions. This section examines the multifaceted applications of RNA in constructing responsive nanostructures that underscore its dynamic role in advancing the field of stimuli-responsive diagnosis and therapeutics.

### Stimuli-responsive fluorescent RNA nanostructures

Conformational changes induced by ligand binding are fundamental for protein allosteric control and are paramount to the regulation of cellular metabolite levels. This concept has been exquisitely adapted into RNA nanostructure design toward the development of RNA-based sensors [100]. Their fundamental architecture is composed of 2 key components, which include a target detection region and a reporter region. The target detection region typically recognizes a specific input molecule and results in the conformational change to the whole structure, which transports the signal to the reporter region and induces to turn on/off the fluorescent signal.

**Fig. 7.**
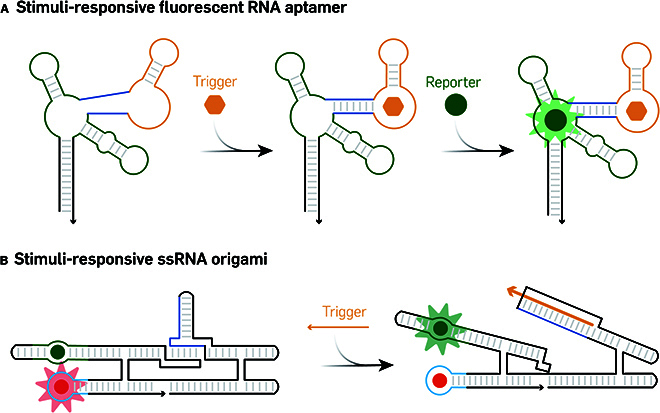
Stimuli-responsive fluorescent RNA nanostructure. (A) Schematic of small molecule responsive fluorescent RNA aptamer. Adapted from Paige et al. [102] with permission. Copyright 2012, AAAS. (B) Design of stimuli-responsive ssRNA origami. The image was adapted under a CC BY-NC license and is attributed to Jespsen et al. [105].

One of the demonstrations of stimuli-responsive RNA nanostructures is based on the fusion of 2 individual aptamers. The detection region is typically an RNA aptamer sensitive to specific targets with a stem–loop structure that remains unstructured until the binding of its target molecule [101]. The reporter region often involves a fluorescent RNA aptamer. These 2 elements are interconnected by a selected linker sequence that forms a stable duplex structure only upon target binding, leading to activation of the reporter [102] (Fig. 7A). This approach has been employed to detect a vast array of small molecules within cells [103], including adenosine, adenosine 5′-diphosphate (ADP), S-adenosylmethionine (SAM), guanine, and guanosine 5′-triphosphate (GTP), combined with different fluorescent colors. Additionally, protein-specific aptamers have been employed for intracellular protein level monitoring [104].

Combined with ssRNA origami design, more stimuli-responsive ssRNA nanostructures can be developed. A Förster resonance energy transfer (FRET) system was developed by integrating 2 FRET-paired fluorescent aptamers into a single-stranded RNA origami scaffold [105] where upon binding of the SAM target molecule, the proximity of the 2 fluorescent aptamers generated a detectable FRET signal, to allow for the detection of cellular SAM levels. The strand displacement can also be applied to induce the conformation change and, thus, decrease the FRET signal (Fig. 7B). More recently, a branched kissing loop was incorporated into the ssRNA origami design to develop a dynamic RNA nanostructure. The outer helices contained 2 branched kissing loops and bent the middle fluorescent aptamer to block its function. Responsive to strand input via loop-mediated strand displacement, the tension within the structure can be released to rescue the fluorescence signal [106].

### Nanostructure-based responsive mRNA manipulation

In nature, mRNAs exhibit complex secondary structures within their UTRs, which is crucial for post-transcriptional regulation of gene expression. However, engineering artificial structures within mRNAs to elicit specific responses to designed inputs remains considerably challenging [107]. In this section, we explore several examples that illustrate the utilization of nucleic acid nanostructure design principles to modulate responsive mRNA expression. Key mRNA components, like the ribosome binding site (RBS), internal ribosome entry site (IRES), and polyadenylation (polyA) tail, can be engineered with tailored sequences, thereby sensitizing the mRNA to specific triggers.

**Fig. 8.**
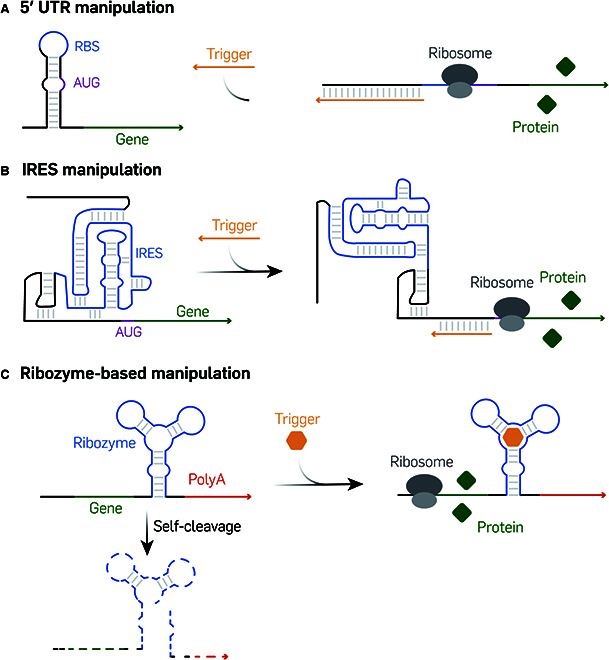
Stimuli-responsive mRNA manipulation. (A) Design of strand displacement mediated RBS regulation. The image was adapted under a CC BY-NC license and is attributed to Simmel et al. [155] (B) Schematic of a Toehold to regulate IRES activity. The image was adapted under a CC BY license and is attributed to Ning et al. [114]. (C) Schematic of ribozyme based polyA tail cleavage for mRNA expression regulation. The image was adapted under a CC BY license and is attributed to Schmidt et al. [156].

The translation initiation of mRNA is intricately linked to the secondary structure in the 5′ UTR. By integrating artificial sequences into the UTR, the 5′ UTR’s secondary structure can be engineered to switch the translation process ON or OFF by leveraging the strand displacement reaction triggered by an input strand [108]. The concept of a strand displacement-mediated mRNA regulator was first proposed in 2004 [109], where the fundamental approach involved the introduction of a *cis*-repressed sequence at the 5′ end of the mRNA. This sequence was used to form a complementary pair with the RBS to create a hairpin structure, thereby hindering the recognition of the RBS by the 30S ribosomal subunit. Next, a *trans*-activating RNA was designed to invade the stem–loop structure through loop-mediated strand displacement to expose the RBS to the ribosome leading to recovery of mRNA translation initiation. Subsequently, a refined design known as the “toehold switch” emerged [110]. This design incorporated the start codon (AUG) into a bulge within the hairpin structure, leaving RBS in the loop. A 12-nucleotide-long toehold sequence was added at the 5′ end to facilitate strand displacement. The invasion of the trigger strand unfolded the hairpin structure and released the start codon and RBS (Fig. 8A). This toehold switch offered not only faster kinetics compared to loop-mediated strand displacement interaction but also eliminated sequence constraints for trigger RNA due to its flexibility in the toehold design. The robustness of this system was demonstrated through the simultaneous testing of up to 12 different orthogonal toehold switches, demonstrating the capabilities of multiplexed regulation. This method was further expanded by incorporating biological circuits to enhance programmability. Here, 2 “switch OFF” systems were developed: the toehold repressor and the 3-way junction (3WJ) repressor [111]. The toehold repressor featured a hairpin structure upstream of the RBS and start codon that can be partially opened by a trigger RNA and form a new hairpin structure that included the RBS and start codon to gene expression. The 3WJ repressor utilized a metastable 3WJ structure that ordinarily allowed expression but can be stabilized by a trigger RNA with the integration of the RBS and start codon into a hairpin that leads to suppression. Later, more elaborate multi-arm RNA junctions were developed to demonstrate this principle further [112], and these innovations could enable the construction of multiple-input logic computing devices that control translation through the addition of trigger RNA strands.

IRESs are special sequences in viral RNA that form secondary structures capable of recruiting eukaryotic ribosomes for translation, independent of the 5′ capping and polyA tail in mRNA. These IRES sequences have been incorporated into mRNA designs, particularly in circular mRNA design, to initiate protein expression, in a number of innovative ways. However, achieving responsiveness and cell specificity in IRES-based mRNA designs remains a major challenge. To address this, a novel RNA module termed “eToehold” was developed to regulate the translation of reporter genes in the presence of specific trigger RNA sequences [113]. The translation efficiency of the coding region in IRES-based mRNA was intricately linked to the secondary structure of IRES, which recruited eukaryotic translation initiation factors (eIFs). In the eToehold system, a short RNA segment complementary to part of the IRES sequence was inserted, resulting in the rearrangement of the stem–loop structures at those sites, resulting in a diminishment of the translation initiation capability. The introduction of a trigger RNA strand that was complementary to the inserted sequence could then be used to displace the obstructive mRNA strand, thereby restoring its protein expression function (Fig. 8B). Another innovative approach, known as Programmable miRNA-responsive IRES Translation Activation and Repression (PROMITAR), was recently introduced [114]. This method involved the incorporation of toehold, stem–loop, and 3WJ structures into the IRES to facilitate the design of complex logic gates. In this system, a complex comprising miRNA and Argonaute (Ago) proteins served as the trigger. This complex bound to specially designed miRNA binding sites without inducing cleavage, subsequently altering the secondary structure. PROMITAR enabled the selective recognition of different miRNAs, thereby allowing conditional protein expression in various cell types.

Ribozymes are unique RNA sequences with catalytic capabilities like mRNA splicing and capping. The integration of ribozymes into mRNA sequence designs provides these constructs with diverse functionalities that can profoundly impact mRNA expression [115]. A notable application involved the use of the hammerhead ribozyme [116], recognized for its ability to bind and cleave RNA at specific sites. The hammerhead ribozyme can be placed downstream of the mRNA coding region, just before the polyA tail, and upon executing its self-cleavage reaction, it split into 2 segments. The absence of the protective polyA tail led to rapid degradation of the coding region in the cell, thereby reducing protein expression levels (Fig. 8C). This ribozyme-incorporated mRNA was responsive to antisense morpholino oligonucleotides targeting specific regions, as well as to small-molecule inhibitors. This approach was particularly effective when combined with adeno-associated virus (AAV) vector-based gene delivery systems [117]. Further refinement of this technique has been seen in the fusion of a regulatory hammerhead ribozyme with an aptamer sensor. The binding of a small molecule to the aptamer induced either a strand-displacement or a helix-slipping event that altered the self-cleavage activity of the hammerhead ribozyme. This configuration allowed it to respond to a variety of small-molecule targets and facilitated the design of switchable ribozyme-based mRNA regulatory systems that were capable of switching protein expression ON or OFF [118].

## Challenges and Opportunities

In this review, we have discussed recent advancements in stimuli-responsive DNA and RNA nanostructures (particularly mRNA), and their applications. We focus on the rational design of these nanostructures, constructed using various assembly strategies and incorporating units responsive to diverse triggers. We categorize stimuli-responsive DNA nanostructures into tile-based systems, DNA origami, and supramolecular DNA assemblies. Leveraging the advanced programmability of DNA nanotechnology, intricate and complex dynamic DNA nanodevices in 1D, 2D, and 3D forms have been designed. These devices, either in pure DNA forms or as hybrids that incorporate other components, can be used to construct biomimetic structures. They exhibit highly autonomous and interactive functions, resembling synthetic cells, and are responsive to a diverse array of triggers that include biological stimuli like DNA/RNA strands, ATP, proteins, antigens, and GSH, as well as physical stimuli such as pH, temperature, electrical signal, metal ions, and light. Different from DNA nanostructures, RNA possesses a rich array of functional components itself, affording great potential and design versatility for responsive RNA-based nanostructures. As a pivotal intermediary in the Central Dogma, RNA plays a crucial role in numerous biological processes, offering substantial opportunities for influencing cellular fate through RNA manipulation. Moreover, the synthesis of single-stranded RNA sequences can be efficiently achieved through a straightforward in vitro transcription process. Such versatile, intelligent, self-responsive DNA and RNA nanostructures, along with their combinations, have been engineered for a broad spectrum of biomedical applications, including diagnostics, biosensing, drug delivery, and therapies for immune and cancer treatment.

Despite remarkable progress in the field, the development of stimuli-responsive DNA nanotechnology faces substantial challenges. A notable issue is the complexity of designing intelligent, biomimetic DNA nanostructures capable of autonomously performing complex functions. These structures aim to mimic cellular components, integrate synthetic metabolic pathways, and incorporate genetic circuits to respond autonomously to environmental stimuli, akin to living organisms. Future solutions may involve combining DNA, RNA, and protein engineering. The scalability and mass production of DNA/RNA nanostructures with high purity are critical for their practical application in various fields, including medicine and nanotechnology. The main challenges include the high cost of synthesis and purification, error rates, efficiency in assembling large structures, stability, and storage issues. These factors limit the scalability and mass production of nucleic acid nanostructures. Innovative approaches addressing these challenges include the development of single-stranded nucleic acid nanostructures, considering the numerous strands used in DNA/RNA tile, and origami methods can be reduced to a single strand [119–121]. Additionally, post-processing from long polycistronic strands enables large-scale enzymatic production of nucleic acid nanostructures [122,123]. Rapid production of 3D DNA nanostructures at constant temperature, which yields high efficiency, shows promise for assembly under physiological conditions [124]. Furthermore, scalable methods for nucleic acid nanostructure purification, such as rate-zonal centrifugation [125] and polyethylene glycol purification [126], are being developed to reduce costs. These advancements are paving the way for more efficient and cost-effective production of DNA/RNA nanostructures.

Another major challenge is the reliance on time-consuming TMSD reactions and chemical triggers, which lead to waste accumulation. Marras et al. demonstrated that sub-second reconfiguration of DNA origami structures could be achieved through cation-mediated actuation by modifying DNA origami with multiple short, weakly complementary overhangs. These overhangs hybridized cooperatively in response to cation concentrations, leveraging the collective binding energy of many distributed binding sites to enable rapid and reversible structural transitions. Unlike the prior TMSD mechanism, which relied on a slower strand displacement process, this approach utilized short overhang sequences that were individually too weak to form stable hybridizations at room temperature but collectively formed stable connections at sufficient cation concentrations, allowing the structure to quickly open or close by adjusting the cation levels [127]. Alternative, remote, and non-invasive control methods such as optical (especially infrared), electric, and magnetic approaches could prove more effective.

Additionally, efficient cellular uptake is crucial; most DNA nanoparticles enter cells via endocytosis and remain trapped in acidic, enzymatic late endosomes/lysosomes [128]. Chemical modication, such as disulfide modifications, combined with the cell surface targeting module, might improve cytosolic uptake [129]. Efficient nuclear delivery of stimuli-responsive DNA nanostructures is critical for genetic material or drug delivery that requires nuclear function. For instance, folding ssDNA scaffolds encoding target genes, such as EGFP or multiplexed genes (mCherry and EGFR), into designed 3D DNA origami structures can achieve efficient gene expression post-electroporation, demonstrating potential for gene delivery applications [130]. Additionally, integrating the CRISPR/Cas gene editing system with DNA nanostructures opens new biomedical application pathways [131,132]. Furthermore, functionalizing DNA origami with antibodies against nuclear factors, such as RNA polymerase, can mediate efficient nuclear delivery via piggybacking [133]. Recent advancements in machine learning and deep learning design tools, such as AlphaFold 3, RoseTTAFold2, and RFdiffusion, have remarkably broadened the potential for targeted delivery of stimuli-responsive DNA/RNA nanostructures in therapeutic applications. AlphaFold 3, for instance, enables the accurate prediction of complex structures involving ions, small molecules, nucleic acids, proteins, and modified residues, outperforming previous specialized tools in modeling protein–ligand and protein–nucleic acid interactions, as well as antibody–antigen complexes. Its high-precision modeling capabilities facilitate the design of DNA nanostructure that can interact specifically and efficiently with target biomolecules, thereby enhancing the efficacy and safety of drug delivery systems [134]. Additionally, Bennett et al. [135] have demonstrated that a fine-tuned RFdiffusion network can rationally design de novo antibodies binding to specific epitope on the target, with experimental confirmation via cryo-EM.

Stabilization of DNA nanoassemblies also presents a dilemma; while treatments like protein coating and silica coating can increase stability, they may interfere with the functionalization of the nanostructures [136,137]. New strategies are needed to balance stability with surface addressability. Site-specific programming of the growth of catalytic polynucleotide brush synthesis on DNA nanostructures offers an innovative pathway to increase nuclease resistance without interfering with functional sites [138].

DNA and RNA are naturally occurring, biocompatible molecules vital for biological functions in all living organisms. Despite their inherent biocompatibility, their application in nanotechnology and drug delivery raises several biosafety concerns. For example, contamination with endotoxins (lipopolysaccharides) from Gram-negative bacteria during the preparation of DNA and RNA nanostructures can cause immunogenetic response and systemic toxicity. Effective endotoxin removal methods, such as endotoxin removal columns, should be employed before using DNA/RNA scaffolds in nanostructure assembly [139]. In addition, synthetic or modified DNA and RNA molecules can be recognized as foreign by the immune system. For instance, unmethylated CpG motifs in DNA may activate Toll-like receptor 9 (TLR9), leading to an immune response [140]. These molecules can also be processed by antigen-presenting cells, such as dendritic cells and macrophages, which may then activate T cells and B cells, triggering a specific immune response [141]. The immune response to DNA and RNA nanostructures in vivo is a complex interplay of various factors, including the DNA sequences, chemical modifications, physical properties, purity, administration, dosage, and host-specific factors [142]. To mitigate potential risks, comprehensive strategies include optimizing DNA design to avoid immunogenic sequences, employing appropriate chemical modification to decrease immunogenicity [143], implementing protective surface coatings or encapsulation techniques [144], choosing appropriate administration routes and dosages, and conducting extensive in vitro and in vivo testing. Furthermore, DNA nanostructure exhibits preferential liver and kidney accumulation due to their interaction with the body’s filtration and metabolic systems, raising additional biosafety concerns [145]. To address this, strategies such as optimizing nanoparticle size, employing targeting ligands, and conducing thorough pharmacokinetic studies are essential. Lastly, a deeper understanding of the in vivo behavior of these nanostructures, including their circulation half-life, pharmacokinetics, and clearance, is essential. Addressing these challenges will be pivotal for the advancement and practical application of DNA nanotechnology.

In the field of RNA nanotechnology, beyond the common concerns shared with DNA nanotechnology, there are 3 additional major challenges currently being confronted. (a) The design of RNA nanostructures remains a complex task. Despite several advancements [38,146], a user-friendly design platform is urgently needed, which encompasses all RNA structural and functional motifs and facilitates the ability to align different components easily. (b) Another obstacle for RNA nanostructure design is the accurate prediction of secondary or tertiary structures, given the intricacy of RNA folding. An advanced artificial intelligence program, like AlphaFold [147] for proteins, is necessary to predict the tertiary structure of RNA sequences to ensure that designed sequences align with specified requirements [148]. The recent development of a large language model in protein structure prediction [149] has great potential in RNA structure prediction. (c) The issues of biosafety and biostability are important in the therapeutic application of RNA nanostructures. The formation of duplex RNA structures can trigger strong immune responses mediated by TLRs. Additionally, the inherent chemical and biological instability of RNA, stemming from its susceptibility to nuclease attack and the presence of a hydroxyl group at the 2′ position, poses considerable challenges. The 2023 Nobel Prize-winning base modifications may offer a solution by incorporating modified bases like pseudouridine and 5-methylcytosine into the RNA nanostructures to decrease the immune response [150]. A parallel method is to modify the backbone, using modification on the ribose to decrease the immunogenicity and increase the biostability of RNA nanostructures [120,151]. However, their effectiveness and safety must be validated through further experimental verification. (d) The development of targeted delivery in RNA nanotechnology is quite limited. One strategy is to transfect plasmid into cells and let cells express RNA nanostructure by itself [100,152]. Another method is to use lipid nanoparticle, which is widely used in siRNA- and mRNA-related therapies [153] and can achieve targeted delivery. Meanwhile, the RNA nanostructures containing functional aptamers can be developed to enable the targeted delivery. For example, an RNA origami fused with lipid aptamer can enable the transmembrane activity of RNA nanostructure. Also, pH-responsive ribozyme can be introduced to enable targeted release around the TME.

In conclusion, this review has explored the sophisticated landscape of stimuli-responsive DNA and RNA nanostructures, highlighting their construction, programmability, and application, especially in biomedicine. These nanostructures represent a nexus of biology and engineering, offering modular and dynamic solutions for diagnostics, therapy, and synthetic biology. Despite remarkable progress, substantial challenges remain in design complexity, scalability, biosafety, efficient cellular uptake, and in vivo stability. Addressing these challenges is essential for realizing the full potential of these advanced materials in practical applications.
